# Olfactory processing in adults with autism spectrum disorders

**DOI:** 10.1186/s13229-016-0070-3

**Published:** 2016-01-19

**Authors:** Bruno Wicker, Elisabetta Monfardini, Jean-Pierre Royet

**Affiliations:** Institut de Neurosciences de la Timone. CNRS & Université Aix-Marseille, Campus Santé Timone 27, Boulevard Jean Moulin, 13385 Marseille cedex 05, France; Integrative, Multisensory, Perception, Action and Cognition Team, Lyon, F-69000 France; INSERM U1028, CNRS UMR5292, Lyon Neuroscience Research Center, Olfaction : From Coding to Memory Team, Lyon, F-69000 France; University Lyon 1, Lyon, F-69000 France; Institut de Médecine Environnementale, Paris, France

**Keywords:** Autism spectrum disorders, Hyperresponsiveness, Olfaction, Suprathreshold detection, Intensity, Pleasantness, Identification

## Abstract

**Background:**

As evidenced in the DSM-V, autism spectrum disorders (ASD) are often characterized by atypical sensory behavior (hyper- or hypo-reactivity), but very few studies have evaluated olfactory abilities in individuals with ASD.

**Methods:**

Fifteen adults with ASD and 15 typically developing participants underwent olfactory tests focused on superficial (suprathreshold detection task), perceptual (intensity and pleasantness judgment tasks), and semantic (identification task) odor processing.

**Results:**

In terms of suprathreshold detection performance, decreased discrimination scores and increased bias scores were observed in the ASD group. Furthermore, the participants with ASD exhibited increased intensity judgment scores and impaired scores for pleasantness judgments of unpleasant odorants. Decreased identification performance was also observed in the participants with ASD compared with the typically developing participants. This decrease was partly attributed to a higher number of near misses (a category close to veridical labels) among the participants with ASD than was observed among the typically developing participants.

**Conclusions:**

The changes in discrimination and bias scores were the result of a high number of false alarms among the participants with ASD, which suggests the adoption of a liberal attitude in their responses. Atypical intensity and pleasantness ratings were associated with hyperresponsiveness and flattened emotional reactions, respectively, which are typical of participants with ASD. The high number of near misses as non-veridical labels suggested that categorical processing is functional in individuals with ASD and could be explained by attention-deficit/hyperactivity disorder. These findings are discussed in terms of dysfunction of the olfactory system.

## Background

Autism spectrum disorders (ASD) are neurodevelopmental disorders that are characterized by poor social communication abilities in combination with repetitive behaviors and restricted interests [1]. ASD is considered a multigenic and multifactorial pathology resulting from interactions between genetic predispositions and environmental risk factors. These interactions impact critical steps in nervous system development.

Extensive abnormal reactions of individuals with ASD to sensory stimuli were highlighted since 1944 by Hans Asperger, especially those concerning touch, smell, and taste. Known people with ASD such as Temple Grandin [2] or Donna Leanne Williams [3] have reported their own vivid experience of unpleasant strong sensation of smell. More recently, parent and clinical reports as well as studies based on sensory profile questionnaires have also demonstrated abnormal responses to odors and tastes among children with ASD [4, 5], but also to cold, heat, pain, tickle, and itch [6, 7]. These accounts describe hyper- as well as hypo-sensitivity to sensory stimuli [8] and suggest a dysfunction of perceptual processes that have yet to be better understood in the olfactory domain. Studies evaluating various olfactory abilities in ASD have been limited and have mainly explored abilities of odor detection (at the threshold level) and identification. On the one hand, studies on odor sensitivity have yielded inconsistent results, with individuals with ASD exhibiting impaired [9], intact [10–12], or increased olfactory sensitivity [13]. These inconsistent findings are likely due to methodological differences. In the study led by Dudova et al. [9], participants were younger than in other studies, had undergone more nasal operations (adenoidectomy) and were taking more medications (e.g., antipsychotics) than controls. However, adenoidectomy has been reported to improve olfactory sensitivity [14], and a pharmacological treatment with antipsychotics could be a potential confounder [15], a point also underlined by Dudova et al. [9]. Thus, an impaired sensitivity in individuals with ASD remains open to question. On the other hand, impaired odor identification performances have consistently been reported [10, 11, 13, 16], although a few differences in results were noted. For example, comparisons of the performance of autistic individuals with that of individuals with Asperger syndrome have revealed that olfactory identification is impaired in autism but not in Asperger syndrome [10, 17]. Galle et al. [10] emphasized that autistic patients display speech delays and a reduced ability to use verbal labels. By contrast, Suzuki et al. [11] reported that participants with Asperger syndrome also demonstrated impaired olfactory identification. Moreover, odor identification performances have been correlated with self-ratings of empathy [18], one of the most clearly impaired cognitive functions in individuals with ASD [19, 20].

Because all studies but one report normal or increased olfactory sensitivity in individuals with ASD, it can be suggested that impaired odor identification performance primarily results from a dysfunction of perceptual representations. Perceptual processes include all sensory processes that follow detection of an event and precede but also contribute to semantic processing of information. In olfaction, these processes are commonly investigated using mainly discrimination and recognition memory tests [21]. For discrimination, no between-group differences in performance [10] or statistical trends [15] have been observed. For recognition memory, to our knowledge, only one study has been performed revealing reduced recognition performances [22]. Thus, very few studies have investigated functionality of perceptual processes in ASD.

In cognitive psychology, it is reported that the incoming sensory stimuli are analyzed at different levels, ranging from superficial (low), sensory analyses (perceptual) to deep (high, that is semantic and cognitive) analyses involving meaning, access to stimulus name, and a variety of associated information [23]. From these concepts, we previously hypothesized that the ratings of intensity, pleasantness, familiarity, and edibility are different olfactory judgments that require activation from perceptual to semantic representations [24, 25]. This assumption was supported by functional neuroimaging data demonstrating the involvement of distinct neural networks (in terms of both structure and hemispheric specialization) in these olfactory judgment tasks [24, 26–28]. Emotion and pleasantness judgment are primary facets of olfaction [29], and the pleasantness judgment is commonly used to rate subjective emotional experience [30–32]. These olfactory tests have also been used in neuropsychological and neuroimaging studies to explore specific deficits in olfactory function in Alzheimer’s and Parkinson’s disease and in patients with schizophrenia or temporal lobe epilepsy [33–37].

In the present study, we used a modified version of our set of olfactory tests. Because abnormally intense and/or unpleasant reactions were reported in literature [4, 6] and because emotional deficits are a core symptom of ASD [38], we focused our tests on odor intensity and pleasantness judgments. Only two recent studies have assessed intensity and/or pleasantness judgments in patients with ASD. Galle et al. [10] did not observe any difference between participants with ASD and control participants, and Hrdlicka et al. [39] observed a difference only for two of 16 odors. We also investigated low-level olfactory function by measuring performances of odor/no odor detection, but by presenting odors at a suprathreshold concentration. Olfactory sensitivity was not assessed, but we could identify errors or bias of participants in relation to their expectation, motivation, and strategy by analyzing data in the framework of signal detection theory [40]. Specifically, bias measurement allows to assess the decision rule adopted by the participants when they are uncertain as to whether an odor is present. Correlatively, errors influence discrimination performances, and it can be hypothesized that people with ASD and controls will show different behaviors, possibly due to attentional deficits [41]. Finally, we used an identification task, but further attempted to distinguish performance as a function of the quality of labels selected by participants [42]. We hypothesized that individuals with ASD would make modified intensity and pleasantness judgments, perceiving odors more intense and/or more unpleasant than control participants. They would display a greater number of errors compared to control participants in the suprathreshold detection task because they would adopt a more liberal decision criterion than the control participants, and would have reduced identification performances.

## Methods

### Participants

The ASD group included 15 adults with high-functioning autism or Asperger syndrome [11 men and 4 women, mean age = 26.3, standard deviation (SD) = 6.0]. They were recruited from all over France through a call for volunteers sent to Centers for Autism Resource (CRA), association of parents, and various psychiatrists or psychologists experts in the field of ASD. The participants were provisionally included in the study if they had received a diagnosis of autism or Asperger syndrome from a psychiatrist or a licensed clinical psychologist. Actual participation required that this diagnosis be recently confirmed, with each participant meeting the criteria for ASD within the past 3 years, according to the revised fourth edition of the *Diagnostic and Statistical Manual of Mental Disorders* [1]. Intelligence quotient (IQ) scores were measured using the third edition of the Wechsler Adult Intelligence Scale and mean IQ score was within the normal range (mean = 99.1, SD = 16.9, range = 77–129). All participants in the ASD group were free of medication and did not suffer from any mental or neurological disorder other than ASD. All but three participants with ASD also participated in a previously published neuroimaging study [43]. Participants in the ASD group were matched by age and gender to a group of 15 typically developing individuals (mean age = 27.8, SD = 9.5; 11 men and 4 women). None of the comparison participants had any neurological or psychiatric disorders. Additional exclusion criteria for participants in both groups included possible brain damage, major medical problems, current substance abuse, and known anosmia or rhinal disorders (e.g., colds, active allergies, asthma). Written consent was obtained from all participants after the procedure was fully explained. The study was approved by the Comité de Protection des Personnes Sud-Méditerrannée I and was conducted in accordance with the Declaration of Helsinki.

### Stimuli

Odorous products were contained in 15-ml yellow glass jars with polyethylene screw lids (Fisher, Elancourt, France). The jars were opaque to mask any visual cues of the odor identity. The odorants were diluted in mineral oil (Sigma-Aldrich, Saint-Quentin-Fallavier, France), and 5 ml of odorous solution (1 %) was prepared and absorbed by the compressed filaments of polypropylene. Because tetrahydrothiophene, acetic acid, and ether released a strong odor, they were diluted 1000 times. The odorants were stored in a refrigerator when not in use and allowed to reach room temperature prior to initiating the experiment. The odorants were supplied by the French companies Givaudan, International Flavor and Fragrance, Perlarom, Lenoir and Davennne, and by a chemical product manufacturer (Sigma-Aldrich, Saint-Quentin-Fallavier, France). For non-odorous stimuli, identical 15-ml yellow glass jars containing compressed filaments of polypropylene and mineral oil only were used.

### Experimental room

The experiment was conducted in a quiet room of 18 m^2^ (4 × 4.5 m). In order to eliminate any undesirable odor, the experimental room was systematically ventilated between the different tests performed for each participant, or between participants. From one to three participants were tested per day. During the procurement phase, the temperature was kept between 20 and 22 °C.

### Procedure

The entire experiment comprised four successive tests: suprathreshold detection, intensity judgment, pleasantness judgment, and identification. Instructions concerning the task were given to each participant immediately before each test. The order of the tests was identical for all participants. In each test, the experimenter presented a jar containing an odorant under the participant’s nose in 30-s intervals, with each odorant presented for approximately 5 s. The entire experiment lasted approximately 30 min. Procedural details are given in our previous papers based on the use of the same olfactory tasks [33–35, 37, 44].

#### Suprathreshold detection test

This test included 24 trials in which the participants were required to decide whether the stimuli were odorant or non-odorant (NO). They responded orally “yes” or “no,” and their answers were recorded by the experimenter. The stimuli were presented in a fixed pseudo-random order and were identical for all participants: tarragon, NO, NO, melon, NO, NO, basil, NO, turpentine, lily, NO, NO, cypress, parsley, tomato, NO, tobacco, cumin, NO, celery, NO, NO, lime, NO.

#### Intensity and pleasantness judgment tests

To limit interactions between perceptual and semantic processes, the intensity and pleasantness judgment tests were successively performed using two sets of the same 12 odorants: rose, caramel, tar, banana, onion, vanilla, camphor, guaiacol, anise, cyclohexane, tomato, and bitter almond. The participants judged to what extent they perceived the odors as intense (intensity test) or pleasant (pleasantness test) using a segmented linear rating scale ranging from 0 to 10. The extremities of the intensity and pleasantness scales were “very weak” and “very strong” and “very pleasant” and “very unpleasant”, respectively.

#### Identification test

To prevent an influence of the intensity and pleasantness judgments on identification performance, the identification test was performed using 12 different odorants (Table 1) than those used for the intensity and pleasantness judgment tests. The participants were asked to identify each odor by choosing a name among a written list of five alternatives that included the veridical label, one name evoking a similar odor, and three names evoking more distinct odors.

**Table 1:** List of odors and list of their respective close and far alternative proposals used in the identification test

Number	Veridical name	Chemical name	Dilution in %	Distractor names			
				1	2	3	4
1	Mushroom	1-Octen-3-ol	1	Mold	Camphor	Liquorice	Lilac
2	Lemon	Mixture	1	Hyacinth	Grapefruit	Vanilla	Apricot
3	Vinegar	Acetic acid	0.1	Orange	Mustard	Gardenia	Cider
4	Lavender	Mixture	1	Incense	Caramel	Mothballs	Thyme
5	Citronella	Mixture	1	Banana	Lychee	Tar	Verbena
6	Clove	Eugenol	1	Lawn	Garlic	Chocolate	Cinnamon
7	Ether	Diethyl ether	0.1	Chloroform	Lily	Pizza	Nail varnish
8	Strawberry	Mixture	1	Biscuit	Raspberry	Petrol	Passion fruit
9	Gas	THT	0.1	Oil	Carnation	Cheese	Turpentine
10	Mint	Mixture	1	Bitter almond	Rose	Liquorice	Anise
11	Pine	Mixture	1	Eucalyptus	Wax	Tobacco	Gingerbread
12	Smoked salmon	Mixture	1	Prawn	Ham	Glue	Jonquil

*THT* tetrahydrothiophene

### Quantitative and statistical analysis

#### Suprathreshold detection data

Detection performance was assessed using the parameters of signal detection theory [40, 45]. By combining the experimental condition (odor or no odor) and the participant’s behavioral response (correct or incorrect), four outcomes were scored. If the stimulus was an odor and the participant declared to have perceived an odor, a “hit” was scored. If the participant did not perceive an odor, a “miss” was scored. If the stimulus was “no odor” and was declared so by the participant, a “correct rejection” was scored. If the participant incorrectly declared to have perceived an odor, a “false alarm” (FA) was scored. From the hit and FA scores, four parameters were then calculated for each participant: the hit rate (*HR*), FA rate (*FR*), discrimination measurement *d’*_*L*_, and bias response (*C*_*L*_). Corwin [46] previously described these calculations in the framework of different paradigms of yes-no tasks as follows:

$$ \begin{array}{c}\hfill d{\hbox{'}}_L= \ln \frac{HR\left(1-FR\right)}{FR\left(1-HR\right)}\hfill \\ {}\hfill {C}_L=0.5\times \ln \frac{\left(1-FR\right)\left(1-HR\right)}{\left(HR\times FR\right)}\hfill \end{array} $$

where *HR* represents the hit rate [(Hit + 0.5) / (*N*_1_ + 1)], *FR* represents the FA rate [(*FA* + 0.5)/(*N*_2_ + 1)], and *N*_1_ and *N*_2_ represent the number of trials with odor and no odor, respectively, for which the participants provided an answer. The discrimination (*d’*_*L*_) score may be good or poor (positive and negative values, respectively). The response bias (*C*_*L*_) scores establish three individual attitudes. The participants may be conservative (tending to respond no to an odor), neutral (responding yes or no with equal probability) or liberal (tending to respond yes), denoted by positive, neutral, or negative values of *C*_*L*_, respectively [47]. One-way analyses of variance (ANOVA) were used to compare between-group performances. The normality of the samples and the homogeneity of their variance were assessed using the Lilliefors [48] and Hartley [49] tests, respectively.

#### Intensity and pleasantness data

The scores obtained for intensity and pleasantness were directly deduced from the values selected for each odor by the participants on the rating scales. Two-way ANOVAs (group x odorants) with repeated measurements on the second factor were performed to separately analyze the scores as a function of groups and odorants. The differences between pairs and groups of means were assessed using multiple orthogonal comparisons [50]. The normality of the samples and the homogeneity of their variance were controlled as indicated above.

#### Identification data

The odor identification scores were determined by attributing to the participant’s response the value 1 when he chose the veridical label and the value 0 when he chose one of the four other alternative names (Table 1). These dichotomous data can be analyzed using the *Q* statistic [51]. However, Cochran [51] has also demonstrated that the *F* statistic, which is computed by analyzing the data as if the measurements were normally distributed variables, yields probability statements that are relatively close to those obtained using the *Q* statistic. Therefore, the data in the present study were analyzed by applying two-way ANOVA with repeated measurements, as indicated by Winer et al. [49]. Second, the identification scores were further analyzed to take into account the quality of the label selected by the participants. Two types of non-veridical labels have been defined [42]. Near misses are names of substances similar to and possibly confusable with test substances, such as moldy for mushroom, grapefruit for lemon, mustard for vinegar, raspberry for strawberry, or petrol for gas. Thus, near misses belong to the same category as the veridical label. By contrast, far misses are clearly incorrect: nail varnish for ether, jonquil for smoked salmon, or hyacinth for lemon. The veridical labels, near and far misses were arbitrarily coded 1, 0.5, and 0, respectively, for each odor and each participant. The respective sums of scores for the veridical labels, near and far misses were computed for each participant, then for all participants of each group. Non-parametric analyses were then performed to compare distributions between both groups. Non-parametrical tests such as the *G* test based on the chi-squared metric and tests based on ranks such as the Mann-Whitney *U* test [48] were used*.*

#### Software for statistical analyses

Analyses were conducted using the Statistica 7.1 (StatSoft®, Inc, Tulsa, OK, USA) and StatView 5.01 (SAS, Institute Inc., Cary, NC, USA) Software Packages for Windows, depending on the type of analyses. Computations for data of the signal detection theory and the Lilliefors test were performed using homemade software written in Quick Basic.

## Results

### Olfactory performance

All participants provided the required ratings for all tests, except for one participant with ASD for identification scores. The arithmetic means of the scores for intensity, pleasantness, and identification were computed as a function of the participant groups (ASD vs. control) and of the odorants.

#### Suprathreshold detection test

**Fig. 1:**
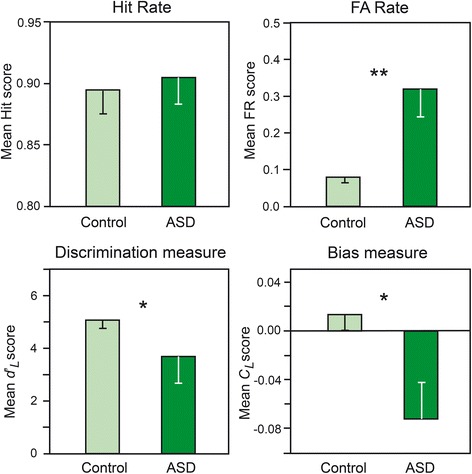
Suprathreshold detection. Representation of Hit rates (HR), FA rates (FR), and discrimination (*d’*_*L*_), and bias (*C*_*L*_) measures as a function of group (control vs. ASD). The vertical bars represent the standard errors of the mean.*p < 0.05; **p < 0.01. *ASD* autism spectrum disorders, *FA* false-alarm

**Fig. 2:**
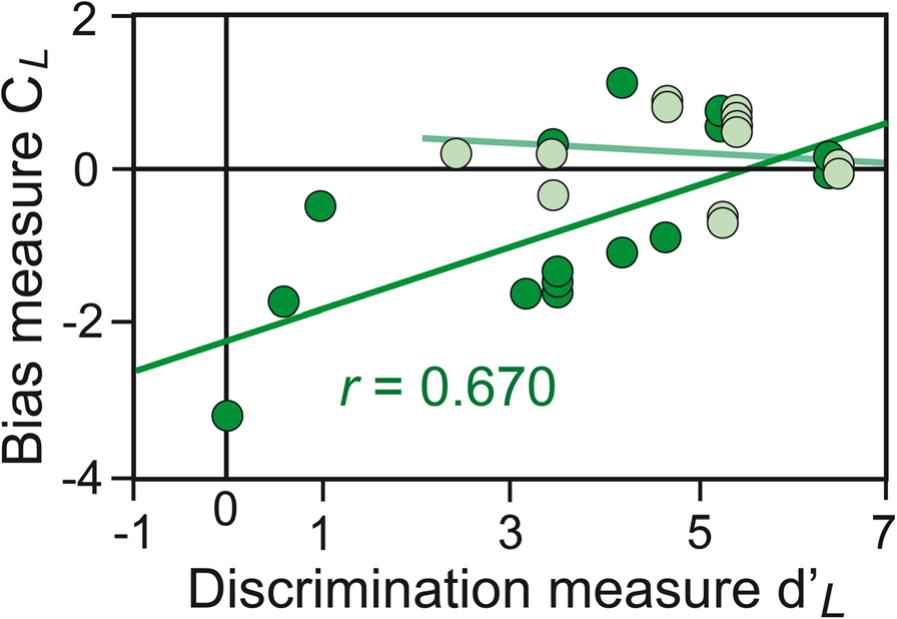
Discrimination (*d*’_*L*_) and bias (*C*_*L*_) measures. *d’*_*L*_ and *C*_*L*_ were positively correlated in ASD patients (in *dark green*) but not correlated in control participants (in *light green*)

The mean Hit and FA scores and the discrimination (*d*’_*L*_) and bias (*C*_*L*_) measures are presented in Fig. 1. The distributions were normal for Hit, FA, *d’*_*L*_ and *C*_*L*_ (*T*_Hit_(15) ≤ 0.209, *T*_FA_(15) ≤ 0.263, *T*_*d’L*_(15) ≤ 0.170 and *T*_*CL*_(15) ≤ 0.137, *p’s* < 0.01) and the homogeneity of their variance was respected [*F*_Hit_(1,14) = 1.233, *F*_FA_(1,14) = 5.830, *F*_*d’L*_(1,14) = 2.599 and *F*_*CL*_(1,14) = 5.148, *p’s* < 0.05]. ANOVAs revealed that the FA scores were significantly higher in the ASD group than the control group [*F*(1, 28 = 9.77, *p* = 0.004] and that the *d*’_*L*_, and *C*_*L*_ scores were significantly lower in the ASD group than the control group [*F*(1, 28) = 5.49, *p* = 0.027 and *F*(1, 28) = 6.86, *p* = 0.014, respectively]. In other words, participants with ASD had lower discrimination scores and displayed more liberal bias than control participants. No significant difference in the Hit scores was identified [*F*(1, 28) = 0.122]. We also observed that the number of Hits was significantly higher than the probability of giving a random response (*p* = 0.5) in the control participants and participants with ASD [*t*(14) = 20.07 and *t*(14) = 18.55, respectively, *p*_*s*_ < 0.001]. Because the bias and discrimination measures are independent [46], we assessed the relationship between these scores by performing linear regression analyses of the control participants and participants with ASD. We determined that the *d*’_*L*_ and *C*_*L*_ scores were positively correlated in the participants with ASD [*r* = 0.670, *F*(1, 13) = 10.56, *p* = 0.006] but not in control participants (*r* = 0.153) (Fig. 2). These results mean that lower discrimination scores in participants with ASD were concomitant with lower (i.e., more liberal) bias scores.

#### Intensity and pleasantness judgment tests

**Fig. 3:**
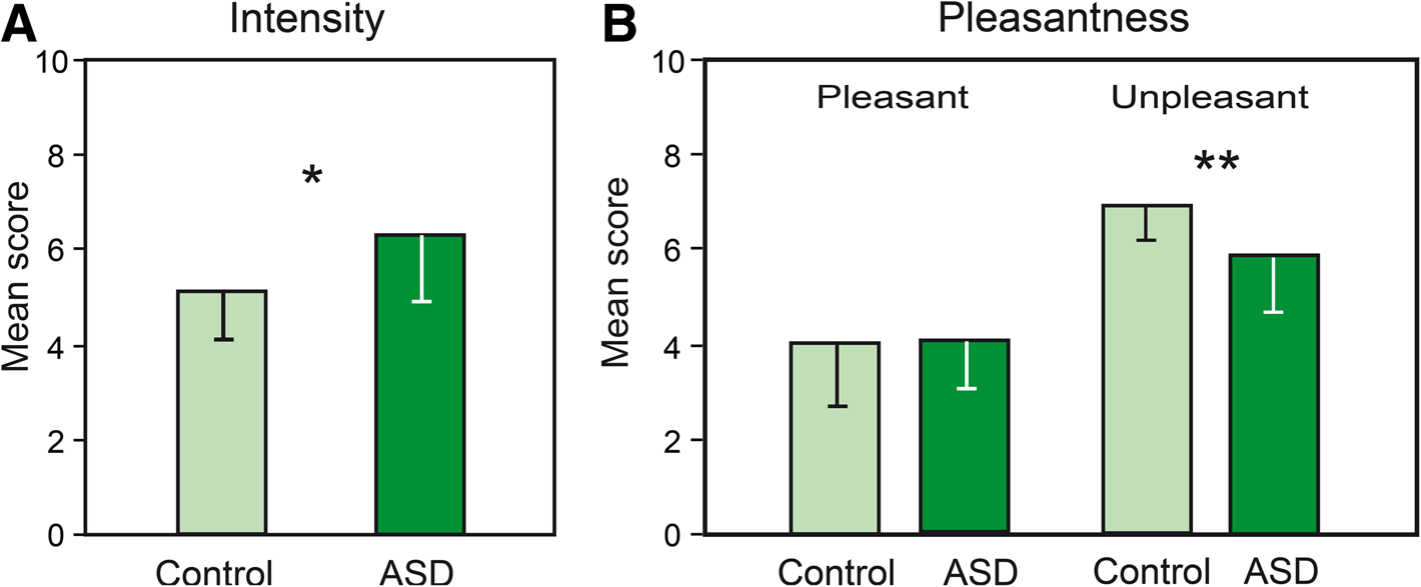
Intensity and pleasantness. Mean intensity scores (**a**) and pleasantness scores (**b**) (0: pleasant; 10: unpleasant) for unpleasant odors in controls and participants with ASD. The vertical bars represent standard errors of the mean. *p < 0.05; **p < 0.01. *ASD* autism spectrum disorders

The distributions of samples were normal (*T*(15) ≤ 0.252, *p’s* < 0.01) and their variances were homogeneous in both groups [controls: *F*(1,14) = 3.51; ASD: *F*(1,14) = 5.81; *p’s* < 0.05]. Two-way ANOVAs with repeated measurements revealed a significant effect of the group factor for intensity scores [*F*(1, 28) = 6.040, *p* = 0.020] (Fig. 3a), participants with ASD displaying higher intensity scores than control participants. No significant effect of the group factor was found for pleasantness scores [*F*(1, 28) = 2.38]. Significant effects of the odor factor were observed for both tasks [*F*(11, 308) = 10.88 and *F*(11, 308) = 22.83, *p*_*s*_ < 0.0001, respectively], but no significant group x odor interactions [*F*(11, 308) = 0.76 and *F*(11, 308) = 1.33, respectively] were observed.

Because the pleasantness task has a bipolar dimension [52], the calculation of the mean scores for the 12 odorants could suppress or reduce between-group differences. Therefore, data for pleasant and unpleasant odorants were analyzed separately. Odorants were distributed into two sets as a function of whether they were judged as being a priori pleasant (rose, caramel, banana, vanilla, anise, bitter almond) or unpleasant (tar, onion, camphor, guaiacol, cyclohexane, tomato), and from measures determined in a previous study [pleasantness = 4.36; unpleasantness = 6.65; *t*(1, 10) = 3.363, *p* = 0.007] [24]. The distributions of samples were normal (*T*(15) ≤ 0.254, *p’s* < 0.01) and their variances were homogeneous in both groups [controls: *F*(1,14) = 5.96; ASD: *F*(1,14) = 3.50; *p’s* < 0.05]. A two-way ANOVA (group x valence) revealed a significant valence effect [*F*(1, 28) = 71.48, *p* < 0.001] due to higher scores for unpleasant than pleasant odors, and a just significant group x valence interaction [*F*(1, 28) = 4.00, *p* = 0.055] due to lower scores (more neutral) for unpleasant odors in the ASD group than in control participants (*p* = 0.019; Fig. 3b). When we analyzed intensity scores by distinguishing both dimensions (pleasant and unpleasant), we found a significant effect of group [*F*(1, 28) = 6.04, *p* = 0.020] and valence [*F*(1, 28) = 16.20, *p* < 0.001] conditions, but not significant group x valence interaction [*F*(1, 28) = 0.65]. In other terms, participants with ASD found both pleasant and unpleasant odors less intense than control participants.

#### Identification test

**Fig. 4:**
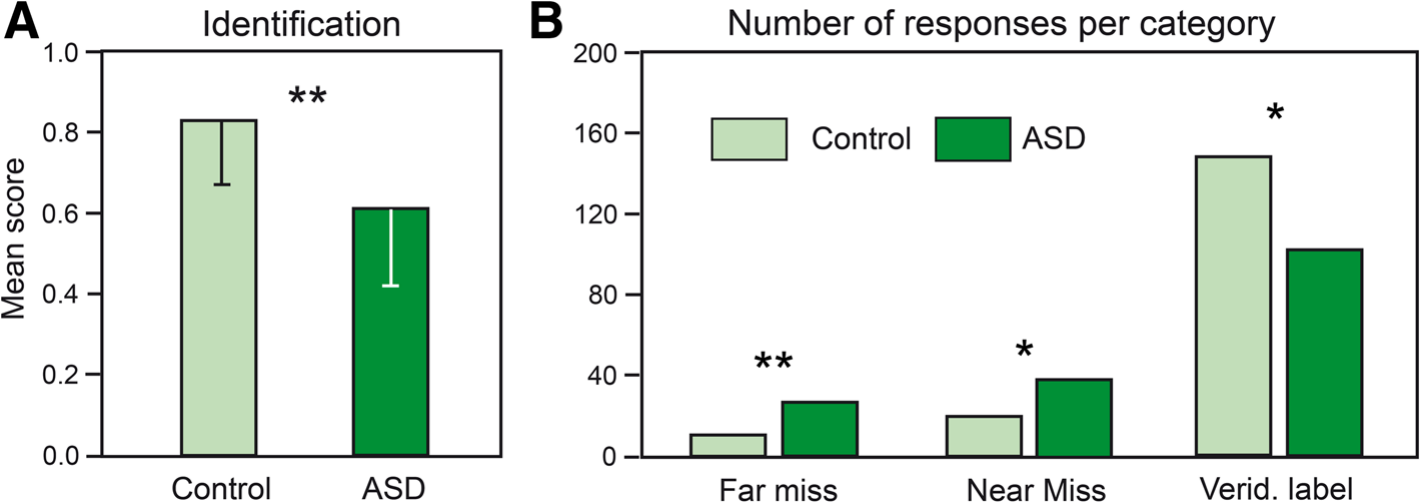
Identification. **a** Mean identification scores and **b** total numbers of far misses, near misses and veridical labels in controls and participants with ASD. The vertical bars represent standard errors of the mean. *p < 0.05; **p < 0.01. *ASD* autism spectrum disorders

For the identification scores, ANOVA revealed significant effects of the group [*F*(1, 27) = 12.06, *p* = 0.002] and odor [*F*(11, 297) = 2.86, *p* = 0.001] factors and no significant interaction between these two factors [*F*(11, 297) = 0.83]. The significant effect of the group factor demonstrates that individuals with ASD were significantly less able to identify odorants (Fig. 4a).

By distinguishing the responses of the participants as a function of the quality of the labels, we calculated the total scores of veridical labels and near and far misses for the participants in each group (Fig. 4b). A *G* test indicated that the two distributions were significantly different [*T*(1, 2) = 20.66, *p <* 0.0001]. The numbers of far and near misses were significantly higher, and the number of veridical labels was significantly lower in participants with ASD than in control participants ([tied *Z* = −2.09, *p* = 0.037; tied *Z* = −2.59, *p* = 0.010; tied *Z* = −2.87, *p* = 0.004, respectively]).

## Discussion

The present study investigated the performance of individuals with ASD in tests exploring a range of low- and high-order olfactory processes. We report three main results: (i) participants with ASD had lower suprathreshold detection scores and used a more liberal decision criterion than the control participants; (ii) participants with ASD judged odors to be more intense and perceived unpleasant odors to be less unpleasant than the control participants; and (iii) participants with ASD identified odors less well than the control participants. Interestingly, incorrect responses in the ASD group included not only far misses but also a higher number of near misses than in the control group.

### Suprathreshold detection

Although most previous studies have suggested that olfactory sensitivity is normal in individuals with ASD [10–12], Ashwin et al. [13] recently demonstrated that olfactory sensitivity to isopropyl alcohol was enhanced in individuals with ASD using a test involving minimal cognitive task demands. Here, we did not rate olfactory sensitivity (at a sub-threshold level), but we examined odor detection abilities at a suprathreshold level for 24 stimuli, including 12 odorants and 12 non-odorants. We observed lower discrimination scores (*d*’_*L*_) in the ASD group compared to the control group, indicating that the ASD participants had difficulty discriminating between the presence and absence of an odor. However, the number of correct detections did not differ between the groups and was clearly higher than what would be expected for a random response. Thus, the low discrimination scores in participants with ASD only resulted from a high number of false alarms: when uncertain, the participants with ASD responded yes more often than no when no odor was present. Consequently, the bias measure further revealed that the participants with ASD had a criterion of more liberal decision than the control participants. The bias and discrimination measures were positively correlated in participants with ASD, that is, lower bias scores (i.e., a more liberal response) corresponded to lower discrimination scores. In other words, when uncertain, individuals with ASD reached the best discrimination performance by adopting a neutral criterion, as did control participants. Liberal bias and poor discrimination in a variety of tasks have been reported in patients with Alzheimer’s, Parkinson’s and Huntington’s diseases [47, 53], and in patients with temporal lobe epilepsy [54, 55]. Liberal bias and poor discrimination have also been observed in healthy participants when the difficulty of the task increases [56, 57]. Thus, a given task can be more difficult for patients to perform than for control participants, and patients consequently adopt a more liberal attitude. The negative response bias exhibited by participants with ASD is likely the product of decisional processes, with no direct bearing upon perceptual detection or discrimination function. Compared with the control participants, the participants with ASD could be impaired in their ability to adapt their decision criterion [53, 58]. Alternatively, the higher number of false alarms may be related to an attentional deficit linked to impulsivity, as ASD is often associated with attention-deficit/hyperactivity disorder [59].

### Intensity and pleasantness judgment

Compared with the control participants, the individuals with ASD judged odors to be more intense. This finding contrasts with that of Galle et al. [10], who did not observe any group differences in average ratings of odor intensity. However, the authors tested only five participants per group, a statistically weak sample size. By contrast, our finding is consistent with previous reports of heightened sensory perceptions in individuals with ASD, regardless of the sensory modality [2–4, 60]. Thus, hyperresponsiveness to sensory stimuli must not be confused with hypersensitivity indicative of a decreased sensory detection threshold. This fundamental distinction is reminiscent of the hyperreactivity to odors that we observed in odor-intolerant migraine patients and that reflects a cortical hyperexcitability [44], whereas most measures of olfactory detection thresholds were not reported to be decreased [61–63]. In these patients, cortical hyperexcitability was evidenced with higher amplitudes of visual and somatosensory evoked potentials [64–66] as well as on findings from magnetic resonance spectroscopy and magneto-encephalography [67, 68]. Similar distinctions were noted for other sensory modalities. For example, hyperacusis to auditory stimuli in ASD is observed in the absence of peripheral auditory abnormalities [69], which suggests difficulties in higher level processing [70]. Blakemore et al. [71] also demonstrated that people with Asperger syndrome are “hypersensitive” to touch at a suprathreshold level (200 Hz), but not at the threshold level (30 Hz). Thus, “hypersensitivity”, which we call hyperresponsiveness, has been observed for different perceptual stimuli and explained for example by enhanced processing of detailed stimuli [72] or impairment in top-down modulation of incoming stimuli [73]. In the present study, hyperresponsiveness could explain the higher intensity rating scores in the ASD group, whereas most previous studies have otherwise indicated normal or reduced olfactory sensitivity [9–12]. At the brain level, animal studies have demonstrated that odor intensity coding initially occurs not only at the level of the olfactory epithelium but also at the level of the olfactory bulbs [74] and piriform cortex [75]. In humans, intensity ratings have been reported to be correlated with activation signals in the medial olfactory areas, including the piriform cortex [76], and to implicate a lateral portion of the inferior frontal gyrus that is associated with semantic processing and is involved in pleasantness and familiarity judgment tasks [26]. The congenital dysgenesis of the olfactory bulbs [77] or dystrophic serotonin axons in the piriform cortex [78] reported in ASD could thus explain the altered judgment of odor intensity in the present study. However, hyperreactivity seems to be more consistent with a dysfunction of the frontal areas involved in higher level processing. Whether hyperreactivity or cortical hyperexcitability can explain the results of the present study could be tested by comparing odor intensity ratings as a function of hyperreactivity measures in participants with ASD.

On average, the participants with ASD judged pleasant odors in a manner similar to the control participants, but they evaluated unpleasant odors to be less unpleasant (i.e., more neutral). These results correspond with the recent observations of Legisa et al. [5] regarding children with high-functioning autism. Galle et al. [10] did not observe any group differences in the average ratings of odor pleasantness, but as noted above, they tested only five participants per group and did not perform separate analyses of a priori pleasant and unpleasant odorants. Testing 16 pleasant and unpleasant odorants, Hrdlicka et al. [39] observed that the participants with ASD rated two pleasant odors as less pleasant. Overall, previous results and our own results suggest that unpleasant odors have a reduced emotional impact in participants with ASD compared to typically developing participants.

Because the participants with ASD in the present study considered odors more intense compared with the control participants and because intensity and unpleasantness scores are typically correlated [24, 79], participants with ASD might be expected to judge unpleasant odors as more unpleasant compared to the judgments of control participants. What explains our somewhat counterintuitive results? We have previously suggested that the amygdala codes emotional intensity [27], i.e., the overall emotional value of an odorant stimulus, as elegantly demonstrated by Winston et al. [80], because it does not code intensity or valence per se but a combination that is observed for emotionally salient (pleasant and unpleasant) odors but not neutral odors. Thus, the impaired amygdala function in individuals with ASD reported in previous studies [43, 81, 82] may explain the abnormal evaluation of unpleasant odors in the present study. In addition, these data may explain why the abnormal evaluation of unpleasant odors in the current study was not paralleled by a reduction of intensity scores. In other words, hyperresponsiveness (hyperreactivity) to stimuli is not incompatible with a flattened emotional reaction.

Because the OFC and the striatum have been implicated in olfactory emotional processes [27, 76, 83–85], particularly when odorants are not highly aversive [86], and during the conscious assessment of the emotional quality of odors [27, 84], a dysfunction of the frontostriatal circuitry in ASD [87–89] could further explain the lower scores in the pleasantness judgment task.

### Identification

We further observed impaired olfactory identification performances in adults with ASD, a result that is consistent with most previous studies [10, 11, 16, 17]. Two studies have reported normal olfactory identification in children with ASD [9, 90], but the discrepancies between studies can be explained by the limited odor identification abilities of the youngest children, which prevents the recognition of deficits until participants are older. An atypical ability to identify odors has been recently suggested to result from a reduced facility to use verbal labels and has been associated with autism rather than Asperger syndrome [10, 17]. However, identification performances are also reduced in Asperger syndrome participants [11], suggesting that this decrease in performance may not be related to the altered role that language plays in the cognitive development of individuals with ASD.

Because categorical processing represents a level of semantic analysis [91], we distinguished near and far misses from incorrect identifications and observed a higher number of near and far misses in the individuals with ASD than in the control participants. This disparity between the two groups is interesting because it differs from the disparity observed between elderly and young persons [92] and between sighted and blind persons [93], among whom only the number of far misses differed. We suggest that the lower identification performance in individuals with ASD is not due to or only to a perceptual or semantic deficit but could also be related to an attentional deficit.

### Limitations of the study

The present study presents several limitations that deserve to be indicated. First, the number of participants in each group was relatively small. Although these numbers are in line with most previous works related to the study of olfactory dysfunction, the present results would deserve to be confirmed by using larger groups of participants. Second, while diagnosis of all ASD patients were given by expert clinicians and thus casts no doubt on their quality, separate measures using the ADOS or ADI-R tools may have been useful to explore the potential effects of symptom severity. Third, our result of higher intensity judgments in ASD interpreted as indicative of a hyperresponsiveness, but not hypersensitivity, should be confirmed by including in a single study odor detection tests at the threshold level and intensity judgment (but also pleasantness) tests at a suprathreshold level. In addition, the high number of false alarms observed here in participants with ASD during the suprathreshold detection test should also prompt to take into account the detection signal theory in such odor sensitivity tests. Fourth, the use of a small number of participants did not allow us to distinguish participants with high-functioning autism and those with Asperger syndrome. Although identification performances have been already investigated in both populations [10, 11], it is not the case of the odor suprathreshold detection test and intensity and pleasantness judgment tests. Fifth, although verbal IQ was within normal range for all participants with ASD, it remains possible that lingual ability has affected performance in the identification task, a point that deserves to be tested. Finally, our control population was not characterized in terms of IQ and was not checked with clinical tests of autistic traits identification. As a consequence, we cannot rule out that some of control participants presented characteristic traits of ASD.

## Conclusions

The present results indicate decreased suprathreshold detection performance in participants with ASD due to poor discrimination and adoption of a liberal decision criterion for their responses. This reduced discrimination performance was concomitant with increased intensity judgment scores for all odors and reduced pleasantness judgment scores for unpleasant odors. Odor identification performance was also impaired in participants with ASD, with an increase in non-veridical labels, including a higher number of far and near misses, compared with control participants. Impaired intensity and pleasantness evaluations were explained by hyperresponsiveness and reduced emotional reaction, respectively, to odors in the participants with ASD. The low identification scores in the participants with ASD were explained not only by far misses but also by a high number of near misses. This result suggests that categorical processing is preserved in ASD and that the near misses could be due to an attentional deficit. While impairments in discrimination and identification could be both related to a domain general attentional deficit not specific to olfaction, differences in intensity and pleasantness ratings could be linked to abnormal functioning of brain areas known to be involved in ASD and in olfaction, such as the amygdala. In brief, by using different olfactory tests, we were able to show dysfunction of different levels of process that were not inevitably specifically olfactory, but that involve several brain regions exhibiting anatomical and functional abnormalities in ASD [94].

## Abbreviations

ASD: autism spectrum disorders
NO: non-odorant
OFC: orbitofrontal cortex
THT: tetrahydrothiophene
